# A phase Ia study of the MEK1/2 inhibitor PD-0325901 with the c-MET inhibitor crizotinib in patients with advanced solid cancers

**DOI:** 10.1038/s44276-025-00133-6

**Published:** 2025-03-26

**Authors:** Peter Gallagher, Christian Rolfo, Elena Elez, Julien Taieb, Jennifer Houlden, Linda Collins, Corran Roberts, Thierry André, Mark Lawler, Federica Di Nicolantonio, Margaret Grayson, Ruth Boyd, Vlad Popovici, Alberto Bardelli, Robbie Carson, Hajrah Khawaja, Pierre Laurent-Puig, Manuel Salto-Tellez, Bryan T. Hennessy, Tim S. Maughan, Josep Tabernero, Richard Adams, Robert Jones, Marc Peeters, Mark R. Middleton, Richard H. Wilson, Sandra Van Schaeybroeck, on behalf of the MErCuRIC Trial Consortium, Peter Gallagher, Christian Rolfo, Elena Elez, Julien Taieb, Jennifer Houlden, Linda Collins, Corran Roberts, Thierry André, Mark Lawler, Margaret Grayson, Ruth Boyd, Vlad Popovici, Alberto Bardelli, Robbie Carson, Hajrah Khawaja, Pierre Laurent-Puig, Manuel Salto-Tellez, Bryan T. Hennessy, Tim S. Maughan, Josep Tabernero, Richard Adams, Robert Jones, Marc Peeters, Mark R. Middleton, Richard H. Wilson, Federica Di Nicolantonio, Vicky Coyle, Francesca Aroldi, Geraldine Perkins, Hans Prenen, Karolien Bettens, Jurgen Delfavero, Sandra Van Schaeybroeck

**Affiliations:** 1 Northern Ireland Cancer Centre, Belfast Health and Social Care Trust, Belfast, UK; 2 Patrick G. Johnston Centre for Cancer Research, School of Medicine, Dentistry and Biomedical Science, Queen’s University Belfast, Belfast, UK; 3 Department of Medical Oncology, University of Antwerp/Antwerp University Hospital, Wilrijk, Belgium; 4 Vall d’Hebron University Hospital and Institute of Oncology (VHIO), Barcelona, Spain; 5 Department of GI Oncology Hôpital Européen Georges-Pompidou, Institut du cancer Paris Carpem, AP-HP, Université Paris Cité, Paris, France; 6 Department of Oncology, Oncology Clinical Trials Office (OCTO), University of Oxford, Oxford, UK; 7 Nuffield Department of Orthopaedics, Rheumatology and Musculoskeletal Sciences, Centre for Statistics in Medicine, University of Oxford, Oxford, UK; 8 Department of Medical Oncology, Sorbonne Université, Hôpital Saint Antoine, Paris, France; 9 Department of Oncology & Candiolo Cancer Institute, University of Torino, Candiolo, TO Italy; 10 RECETOX, Faculty of Science, Masaryk University, Brno, Czech Republic; 11 Department of Oncology, Molecular Biotechnology Center, University of Torino, Torino, Italy; 12 IFOM ETS, The AIRC Institute of Molecular Oncology, Milano, Italy; 13 Centre de recherche des cordeliers, INSERM, Sorbonne Université, Université Paris Cité, Paris, France; 14 Royal College of Surgeons in Ireland University of Medicine and Health Sciences, Dublin, Ireland; 15 Department of Oncology, Old Road Campus Research Building Roosevelt Drive, University of Oxford, Oxford, UK; 16 Department of Molecular and Clinical Cancer Medicine, University of Liverpool, Ashton St, Liverpool, UK; 17 Cardiff University and Velindre University NHS Trust, Cardiff, UK; 18 Agilent Technologies, Genomics, Diagnostics and Genomics Group, Diegem, Belgium

## Abstract

**Background:**

Single-agent MEK1/2 inhibition has been disappointing in clinical trials targeting *RAS* mutant (MT) cancers, probably due to upstream receptor activation, resulting in resistance. We previously found that dual c-MET/MEK1/2 inhibition attenuated *RAS*MT colorectal cancer (CRC) xenograft growth. In this study, we assessed safety of MEK1/2 inhibitor PD-0325901 with c-MET inhibitor crizotinib and determined the optimal biological doses for subsequent clinical trials.

**Methods:**

In this dose-escalation phase I trial, patients with advanced solid tumours received PD-0325901 with crizotinib, using a rolling-6 design to determine the maximum tolerable dose (MTD) and safety/tolerability. Blood samples for pharmacokinetics and skin biopsies were collected.

**Results:**

Twenty-five patients were recruited in 4 cohorts up to doses of crizotinib 200 mg B.D continuously with PD-0325901 8 mg B.D, days 1–21 every 28 days. One in six patients exhibited a dose-limiting toxicity at this dose level. Drug-related adverse events were in keeping with single-agent toxicity profiles. The best clinical response was stable disease in seven patients (29%).

**Conclusions:**

PD-0325901/crizotinib can be given together at pharmacologically-active doses. The MTD for PD-0325901/crizotinib was 8 mg B.D (days 1–21) and 200 mg B.D continuously in a 28-days cycle. The combination was further explored with an alternate MEK1/2 inhibitor in *RAS*MT CRC patients.

**EudraCT-Number:**

2014-000463-40

## Background

Targeting oncogenic drivers has improved outcomes for several human malignancies [1]. Compared with lung or breast cancers, fewer clinically-actionable oncogenic alterations have been identified in colorectal cancer (CRC) [2]. Monoclonal antibodies against the epidermal growth factor receptor (EGFR), such as cetuximab and panitumumab, together with standard chemotherapies form the mainstay of tumour targeted therapy for metastatic *RAS/BRAF* wild-type (WT) CRC, whereas improvements in responses and survival have been found with the BRAF kinase inhibitor encorafenib when combined with cetuximab in *BRAF* mutant (MT) metastatic CRC (mCRC) [3–5].

The small GTPase KRAS is mutated and constitutively active in about 20–25% of all human cancers, in particular pancreatic, lung and colorectal tumours [6]. Exon 2 (codon 12, 13), 3 (codon 59, 61) and 4 (codon 117, 146) KRAS and NRAS mutations occur in 50–55% of CRC patients [7], where they have been associated with resistance to EGFR-targeted therapies and poor survival [8, 9]. Despite many efforts, RAS has proven difficult to target in CRC. Covalent inhibitors targeting KRAS (G12C), present in approximately 3% of CRC patients [10], in combination with other drugs are in phase 1-3 clinical trials development [11] whereas targeted approaches of the more common KRAS mutations (e.g., G12D) and pan-(K)-RAS inhibitors are at preclinical/first-in-human stages in pan-cancer models [12, 13]. Attempts to target the key single effectors downstream to KRAS (e.g., MEK1/2 and phosphatidylinositol 3-kinase [PI3K]) revealed only modest or no efficacy [14–16]. The success of MEK1/2 inhibitors as monotherapy has been limited by rapid activation of feedback loops or crosstalk with other pathways, including the PI3K-AKT pathway [17]. We previously identified cMET-dependent activation of STAT3 as a key mediator of resistance to MEK inhibitors in *KRAS*MT CRC in vitro and in vivo. Additionally, pharmacological blockade of this resistance pathway using the cMET inhibitor crizotinib (formerly PF-02341066) increased MEK1/2-inhibitor-induced apoptosis and growth inhibition in vitro and in vivo in *KRAS*MT CRC models [18]. Based on this and other studies [19], we reasoned that MET could represent a valuable therapeutic target when combined with MEK1/2 inhibitors in patients with *KRAS*MT mCRC, and selected the combination of crizotinib and the MEK1/2 inhibitor PD-0325901 for clinical investigation.

PD-0325901 is a highly potent, selective, non-ATP-competitive oral small molecule inhibitor of both MEK1 and MEK2 [20]. A phase I study of PD-0325901 in patients with advanced malignancies defined the maximum tolerated dose (MTD) as 15 mg twice daily (B.D) continuously, but this was subsequently revised due to the late occurrence of retinal vein occlusion (RVO) [21, 22]. The second drug, crizotinib, is an oral ATP-competitive small-molecule inhibitor of c-MET, ALK and ROS1 [23]. Crizotinib is approved for the first-line treatment of patients with metastatic lung cancer whose tumours are either ALK or ROS1 positive [24, 25], and the recommended oral dose is 250 mg B.D.

We conducted a multicentre single-arm dose-escalation study of crizotinib with PD-0325901, to establish the MTD and assess the safety and toxicity profile for this combination in patients with pre-treated advanced solid tumours. Secondary objectives included the characterisation of the regimen’s pharmacokinetics (PK) profile, analysis of pharmacodynamic (PD) biomarkers and anti-tumour activity, and to define the recommended phase II (RPII) dose and schedule for this combination.

## Methods

### Study design and treatments

The study used an open-label, dose-escalation, rolling six design [26] and was conducted in 4 European centres (United Kingdom, Spain and Belgium) to determine the maximum tolerable dose (MTD), recommended phase II dose (RPII) and safety profile of PD-0325901 with crizotinib, as well as the evaluation of pharmacokinetics (PK) and pharmacodymamics (PD) (ClinicalTrials.gov number: NCT02510001).

Collective PK/PD in vivo xenograft modelling and efficacy data for crizotinib has shown that its target-efficacious free plasma concentration range was 8.1 to 12.8 nM (equivalent to 40 to 62 ng/mL total drug in human plasma) for cMET and that this was achieved with a crizotinib dose of 200 mg once daily (O.D) [27, 28]. Hence, crizotinib 250 mg O.D was the lowest dose assessed in this dose escalation study. Initial phase I studies have shown that doses of PD-0325901 > 15 mg twice a day (B.D), irrespective of the schedule, resulted in intolerable toxicity [21, 22]. Additionally, doses ≥2 mg PD-0325901 B.D achieved plasma concentrations required to inhibit MEK1/2 in xenograft studies (16.5–53.5 ng/mL) [29] and consistently caused ≥60% suppression of pERK1/2 in melanoma patient samples [21]. Hence, doses of 2–8 mg B.D of PD-0325901 were evaluated in this dose escalation study.

The study design consisted of 4 pre-defined dose levels (Supplementary Fig. S1A, B), starting at crizotinib 250 mg O.D with PD-0325901 2 mg B.D, then crizotinib 200 mg B.D with PD-0325901 2 mg B.D, crizotinib 200 mg B.D with PD-0325901 4 mg B.D. and crizotinib 200 mg B.D with PD-0325901 8 mg B.D. In case of defining dose levels 2 or 3 as MDT, an intermediate dose level was permitted (Supplementary Fig. S1A), dependent on the emergent clinical data and dose-limiting toxicities (DLT). Patients initiated treatment with a 7-day run-in period of PD-0325901 alone, followed by PD-0325901 combined with crizotinib for 21-days in a 28-day cycle. In subsequent cycles, patients received PD-0325901 (days 1–21 every 28 days) with oral crizotinib continuously (Supplementary Fig. S1C). The MTD was defined as the dose of PD-0325901 and crizotinib below that at which two out of up to six patients experienced a dose-limiting toxicity (DLT). Patients could remain on combination treatment until disease progression or predefined unacceptable toxicity. At each dose level and before escalation to the next level, a safety study board comprising representatives from the study sponsor and collaborating investigators reviewed individual patient safety and DLTs. DLTs were defined as an almost certainly or probable drug-related adverse events to either drug (Supplementary Table S1), during the first cycle of treatment.

### Patient selection

Eligible patients were ≥16 years of age with advanced solid tumours, Eastern Cooperative Oncology Group (ECOG) performance status (PS) of 0 or 1, a life expectancy of >3 months and adequate organ function. Key exclusion criteria included a history of hypoalbuminaemia or the presence of ascites or pleural effusions requiring taps, untreated or unstable brain metastases, a past history of retinal vein occlusion, intraocular pressure >21 mmHg or patients considered at risk of retinal vein thrombosis. Patients were excluded if they had received previous treatment with HGF, c-MET or MEK1/2 inhibitors.

### Safety and efficacy assessments

Safety assessments comprised of physical examination including vital signs, weight, ECOG PS assessment, documentation of adverse events (AEs) and concomitant medication, with regular monitoring of haematology and biochemistry. Ophthalmic examinations (including visual acuity, pressure, perimetry, slit lamp examination, fundoscopy, and optical coherence tomography), ECG and Echocardiography/MUGA were performed at baseline and at pre-defined time-points during the trial. Toxicities were graded according to the National Cancer Institute Common Terminology Criteria for Adverse Events, version 4.03. At this stage, patients were not required to have measurable disease. Anti-tumour activity was assessed by radiological investigations according to revised RECIST, version 1.1, at baseline (within 28 days before day 1 of cycle 1), then every 2 cycles and at the end of treatment.

### Pharmacokinetics

The concentration of crizotinib, PD-0325901 and its metabolite PD-0315209 in the plasma was measured in 22 patients. PK analyses were performed to ensure that the putative target levels of each drug to inhibit p-c-MET and pERK1/2 levels were reached with the combination treatment. Plasma samples for 24-h PK profiles for PD-0325901/PD-0315209 and crizotinib were collected during cycle 1 day -1 and day 21 and during cycle 1 day 21 and 28 respectively. PK trough samples (pre-dose and 2-h post dose) were obtained on day 21 of cycles 2, 4, 6, 8, 10 and 12. Plasma concentrations were determined using a validated high-performance liquid chromatography mass spectrometry (HPLC-MS/MS) assay following solid-phase extraction of the plasma sample. Analyses of plasma samples for PD-0325901/PD-0315209 and crizotinib were performed by Quintile Biosciences (New York, USA) and Covance (Indianapolis, USA) respectively.

### Pharmacodynamics

All patients consented to a fresh frozen punch skin biopsy during screening and on cycle 1 day 15 (±7 days). PD markers of MEK1/2 inhibition (pMEK1/2 and pERK1/2 levels) in skin biopsies were assessed by Western blotting, previously described [18, 30]. Anti-pMEK1/2^S217/221^, MEK1/2, pERK1/2^T202/Y204^ and ERK1/2 (Cell Signaling Technology, Beverly, MA, USA) were used in conjunction with a HRP-conjugated anti-rabbit secondary antibody (Amersham, Buckinghamshire, UK). Western blot images were developed using the G:BOX Chemi XX6 gel doc system (Syngene, Cambridge, UK). Densitometry on Western blot images was performed using ImageJ software. At this stage, patients could also consent to an optional paired tumour biopsy to be performed during screening and on cycle 1 day 15 (±7 days), however within this dose escalation study no paired tumour biopsies were performed.

### Statistical analyses

Safety and efficacy data were summarised using descriptive statistics. Evaluable patients for toxicity were those patients that received at least one dose of one or both drugs. Evaluable patients for MTD or dose escalation were those patients who completed cycle 1 or withdrew early for experiencing a DLT. Response analyses (i.e., RECIST 1.1) were performed on an intention-to-treat basis, and any patient who received any dose of study treatment was evaluable for response. Progression-free-survival (PFS) was defined as the time between receiving the first dose of study medication (cycle 1, day -7) to disease progression or death from any cause. Overall survival (OS) was defined as the time between cycle 1, day -7 to death from any cause. Statistical significance was calculated from distinct technical replicates by Student’s *t* test (2-tailed, 2 sample equal variance on unpaired data), in GraphPad Prism 8. Graphs were plotted as means with error bars represented as SD; statistical significance was denoted as follows: ****=*p* < 0.0001, ***=*p* < 0.001, **=*p* < 0.01, *=*p* < 0.05, ns=*p* > 0.05.

## Results

### Baseline demographics

Twenty-five patients with advanced solid tumours were included in the study. Baseline characteristics of the population are summarised in Table 1. The median age of patients was 63.4 years (range, 36–78), 52% were male and all patients had an ECOG Performance Status (PS) of 0 or 1. Patients were heavily pre-treated and 60% had received ≥3 prior anti-neoplastic regimens. CRC was the most common (52%) solid tumour type.

**Table 1:** Baseline patient demographic, characteristics and treatment allocation.

Characteristics	Dose escalation cohort				
Cohort	Cohort 1	Cohort 2	Cohort 3	Cohort 4	Total
PD-0325901	2 mg B.D	2 mg B.D	4 mg B.D	8 mg B.D	(*n* = 25)
crizotinib	250 mg O.D (*n* = 6)	200 mg B.D (*n* = 5)	200 mg B.D (*n* = 6)	200 mg B.D (*n* = 8)	
Demographics					
Age (years) median (range)	65.8 (36–78)	64.8 (48–69)	58.4 (52–71)	61.2 (36–73)	63.4 (36–78)
Gender					
Male, *n* (%)	2 (33.3)	2 (40)	3 (50)	6 (75)	13 (52)
Female, *n* (%)	4 (66.7)	3 (60)	3 (50)	2 (25)	12 (48)
ECOG PS					
0, *n* (%)	1 (16.7)	2 (40)	1 (16.7)	4 (50)	8 (32)
1, *n* (%)	5 (83.3)	3 (60)	5 (83.3)	4 (50)	17 (68)
Tumour origin					
Hepatobiliary – pancreatic Cancer, *n* (%)	1 (16.7)	1 (20)	0 (0)	0 (0)	2 (8)
Colorectal Cancer (incl. Appendiceal), *n* (%)	2 (33.3)	2 (40)	4 (66.6)	5 (62.5)	13 (52)
Gastric Cancer, *n* (%)	0 (0)	0 (0)	0 (0)	2 (25)	2 (8)
Small Bowel Cancer, *n* (%)	1 (16.7)	0 (0)	0 (0)	0 (0)	1 (4)
CUP (cancer of unknown primary), *n* (%)	1 (16.7)	0 (0)	1 (16.7)	0 (0)	2 (8)
Renal, *n* (%)	1 (16.7)	0 (0)	0 (0)	0 (0)	1 (4)
Ovarian, *n* (%)	0 (0)	1 (20)	1 (16.7)	0 (0)	2 (8)
Cervix, *n* (%)	0 (0)	1 (20)	0 (0)	0 (0)	1 (4)
Lung, *n* (%)	0 (0)	0 (0)	0 (0)	1 (12.5)	1 (4)
Median range of prior systemic therapies					
1–2, *n* (%)	3 (50)	2 (40)	0 (0)	5 (62.5)	10 (40)
3–4, *n* (%)	3 (50)	2 (40)	3 (50)	2 (25)	10 (40)
5–6, *n* (%)	0 (0)	1 (20)	1 (16.7)	1 (12.5)	3 (12)
≥ 7, *n* (%)	0 (0)	0 (0)	2 (33.3)	0 (0)	2 (8)

*ECOG* Eastern Cooperative Oncology Group, *PS* Performance status, *Incl.* including.

### Dose escalation

One out of the 25 patients did not commence study treatment due to disease-related symptoms. Twenty-four patients were enrolled into 4 cohorts according to dose level (Supplementary Table S2). Three patients (2 and 1 patients treated in cohort 3 and 4 respectively) did not complete cycle 1 and were therefore not evaluable for DLTs. No DLTs were observed in patients treated in dose level 1-3 (Cohort 1-3). One DLT of grade 3 fatigue was observed at dose level 4 (cohort 4) (Supplementary Table S2). Dose level 4 (200 mg B.D crizotinib days 1–28, and PD-0325901 8 mg B.D days 1–21) was therefore defined as the MTD.

### Treatment exposure

A total of 51 cycles of treatment were given, with a median of 2 cycles per patient (range, 0–6). The most common reason for discontinuation of study treatment was disease progression (83%), while remaining reasons were disease-related adverse events (8%), toxicity (4%) and investigator decision (4%). At the time of data analysis, no patients remained on study treatment. Following treatment discontinuation, 2 patients received further systemic therapy.

### Safety

There were in total 159 drug-related adverse events (DR-AE), of which 151 were determined to be related to PD-0325901 and 124 related to crizotinib. Common DR-AEs, observed in ≥2 patients, are summarised in Tables 2, 3. The most common drug-related adverse events were rash (83%), followed by nausea (38%), fatigue (33%), diarrhoea (33%), vomiting (29%), oedema (25%), anaemia (21%), hypoalbuminaemia (21%) and oral mucositis (21%) (Table 2; Supplementary Table S3). Twenty-five DR-AEs were observed in the 7 patients treated at the highest dose level (Table 3), with rash being the most common DR-AE (86%). Most DR-AEs were of grade 1 or 2, and there were no grade 4 DR-AEs or deaths due to DR-AEs (Tables 2, 3). The most common grade 3 DR-AEs were fatigue, anaemia and hypoalbuminaemia, observed in 6 patients (25%) (Supplementary Table S4). When evaluated by cohort, there were no clear differences in the incidence of toxicity with increasing dose, although the one incidence of grade 3 abdominal pain occurred at the highest dose level, and the two incidences of grade 3 fatigue at dose levels 3 and 4.

**Table 2:** Summary of treatment-related, non-hematologic and non-biochemical, biochemical and haematological adverse events occurring in ≥2 patients who received treatment (All cohorts).

No. of patients AE affected *n*, (%)	Grade 1 (*n* = 24)	Grade 2 (*n* = 24)	Grade 3 (*n* = 24)	Grade 4 (*n* = 24)
NON-HAEMATOLOGICAL AND NON-BIOCHEMICAL				
Rash	16 (67)	4 (17)	0 (0)	0 (0)
Dry skin	2 (8)	0 (0)	0 (0)	0 (0)
Constipation	2 (8)	0 (0)	0 (0)	0 (0)
Diarrhoea	7 (29)	1 (4)	0 (0)	0 (0)
Nausea	7 (29)	2 (8)	0 (0)	0 (0)
Vomiting	5 (21)	1 (4)	1 (4)	0 (0)
Mucositis (including mouth pain and ulcers)	5 (21)	0 (0)	0 (0)	0 (0)
Anorexia	3 (13)	1 (4)	0 (0)	0 (0)
Fatigue	4 (17)	2 (8)	2 (8)	0 (0)
Oedema	4 (17)	2 (8)	0 (0)	0 (0)
Visual disturbance (including blurred vision)	4 (17)	0 (0)	0 (0)	0 (0)
QTc prolongation	4 (17)	0 (0)	0 (0)	0 (0)
BIOCHEMICAL				
ALP increase	2 (8)	0 (0)	0 (0)	0 (0)
ALT increase	2 (8)	0 (0)	0 (0)	0 (0)
Hypoalbuminaemia	0 (0)	3 (13)	2 (8)	0 (0)
HAEMATOLOGICAL				
Anaemia	1 (4)	2 (8)	2 (8)	0 (0)

**Table 3:** Treatment-related adverse events (AE) experienced by the 7 patients in Cohort 4, treated at the MTD, who started treatment, by CTCAE grade.

No. of patients AE affected *n*, (%)	Any Grade (*n* = 7)	Grade 3 (*n* = 7)	Grade 4 (*n* = 7)
NON-HAEMATOLOGICAL AND NON-BIOCHEMICAL			
Rash	6 (86)	0 (0)	0 (0)
Abdominal pain	1 (14)	1 (14)	0 (0)
Constipation	1 (14)	0 (0)	0 (0)
Diarrhoea	3 (43)	0 (0)	0 (0)
Fatigue	3 (43)	1 (14)	0 (0)
Anorexia	1 (14)	0 (0)	0 (0)
Nausea	1 (14)	0 (0)	0 (0)
Oedema	2 (29)	0 (0)	0 (0)
Paronychia	1 (14)	0 (0)	0 (0)
QTc prolongation	1 (14)	0 (0)	0 (0)
Visual disturbance	1 (14)	0 (0)	0 (0)
Vomiting	2 (29)	0 (0)	0 (0)
BIOCHEMICAL			
LDH increase	1 (14)	0 (0)	0 (0)
HAEMATOLOGICAL			
Thrombocytopenia	1 (14)	0 (0)	0 (0)

Three patients required a dose reduction for PD-0325901 due to DR-AEs, including grade 2 oedema, grade 2 acneiform rash and grade 3 fatigue. No patients received a dose reduction for crizotinib. A total of 9 serious adverse events were reported in 7 patients. However, only one of these (grade 3 hypoalbuminaemia at dose level 3), was thought to be drug-related (PD-0325901), resulting in treatment discontinuation (Supplementary Table S2).

### Pharmacokinetics

**Fig. 1:**
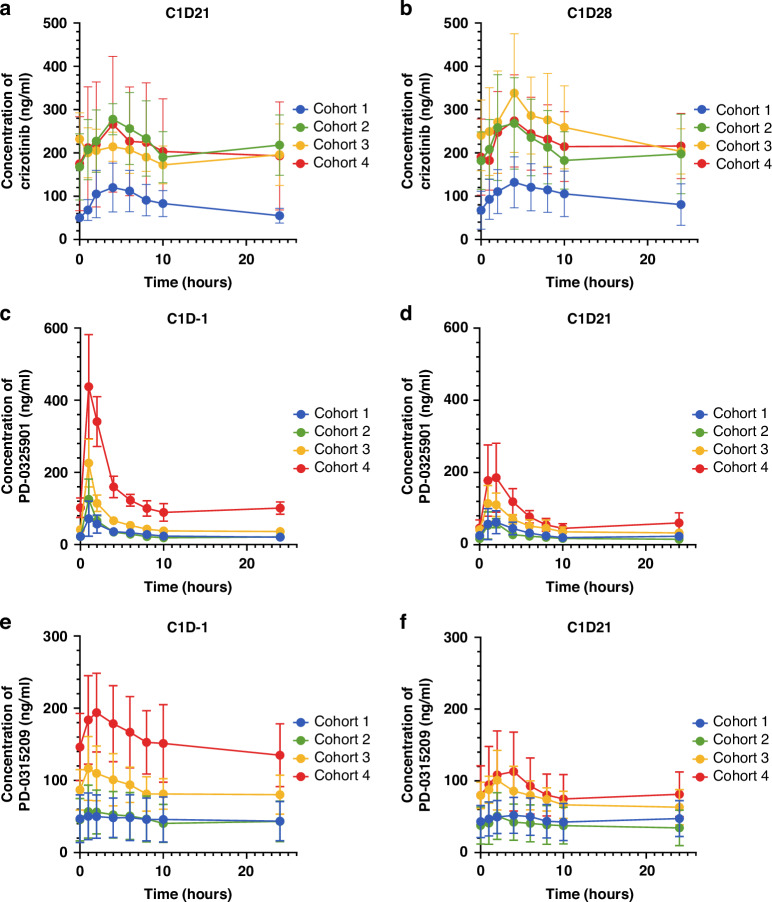
Patients plasma concentrations for crizotinib, PD-0325901 and PD-0315209. Twenty-four hours PK profiles for crizotinib, obtained C1D21 (**a**), C1D28 (**b**), for PD-0325901 obtained C1D-1 (**c**) and C1D21 (**d**) and for PD-0315209 obtained C1D-1 (**e**) and C1D21 (**f**).

The effect of PD-0325901 and crizotinib on PK parameters of crizotinib and PD-0325901 respectively was assessed in 22 patients at the 4 dose levels (Fig. 1; Supplementary Table S5). Following oral administration, crizotinib was absorbed with peak plasma concentrations occurring within 2.76 and 5.21 after dosing, comparable to those observed when administered alone [28]. The T_max_, C_max_, C_min_, AUC_0-10h_, AUC_0-24h_ of crizotinib (measured for 0–24 h and 0–10 h in cohort 1 and 0–10 h only in cohorts 2-4) were similar on Cycle 1 Day 21 and Cycle 1 Day 28, indicating that the multiple dosing had reached steady state by day 21 (Supplementary Table S5). There was no significant difference between the cohorts 1 (O.D) and 2–4 (B.D) for the time taken to reach C_max_. However, the B.D dosing of crizotinib did result in significant (1.6–2.6 fold; *p* < 0.05) increases in C_max_, C_min_ and AUC_0-10h_ in cohorts 2-4 on days 21 and 28 compared with cohort 1. The dose escalation of PD-0325901 in cohort 2 to cohort 4, did not appear to affect the PK parameters of crizotinib.

After oral administration, PD-0325901 was absorbed rapidly, with peak plasma concentrations occurring within 1.02 and 2.27 after dosing, comparable to those observed when administered alone [21]. There was no significant difference in T_max_ for PD-0325901 and its metabolite PD-0315209 between cycle 1 day -1 and day 21. There was no significant difference between cohort 1 and cohorts 2 - 4 in T_max_ for PD-0325901 following an oral dose of PD-0325901 showing that the co-administration of PD-0325901 and crizotinib had little effect on T_max_. The average time (±SD) to reach C_max_ for PD-0325901 on Cycle 1 Day -1 was 2.2 ± 1.9 h for cohort 1 and 1.2 ± 0.4 h for cohorts 2 to 4. For Cycle 1 Day 21, the average time ( ± SD) to reach C_max_ for PD-0325901 was 2.2 ± 1 h for cohort 1 and 1.9 ± 0.8 h for cohorts 2 to 4. In general, the time to reach maximum concentration of the metabolite was similar to the T_max_ for PD-0325901 (1.5 ± 0.5 h for Cycle 1 Day -1 and 3.0 ± 2.1 h for Cycle 1 Day 21 for cohorts 2-4). For cohort 1, the T_max_ for PD-0315209 was numerically later (4.3 ± 3.4 h for Cycle 1 Day-1 and 3.5 ± 1.2 h for Cycle 1 Day 21). For B.D dosing of crizotinib and PD-0325901 (cohorts 2-4) on Cycle 1 Day -1, there was a linear relationship between the dose of PD-0325901 and the AUC_0-10h_, C_max_ and C_min_. Interestingly, the AUC_0-10h_, C_max_ and C_min_ for PD-0325901 and PD-0315209 were lower on Cycle 1 Day 21 than on Cycle 1 Day -1 especially following a 8 mg B.D dose of PD-0325901 (Supplementary Fig. S2A–C). Furthermore, the pre-dose concentrations of PD-0325901 and PD-0315209 were lower on Cycle 1 Day 21 and Cycle 2 Day 21 than on Cycle 1 Day -1, in particular at a dose of 8 mg B.D of PD-0325901 (Supplementary Fig. S2D). These data would indicate that co-administration of crizotinib with PD-0325901 appears to reduce the plasma concentration of PD-0325901 and its metabolite on Cycle 1 Day 21 compared to Day -1, especially at the highest 8 mg B.D dose.

### Pharmacodynamics

**Fig. 2:**
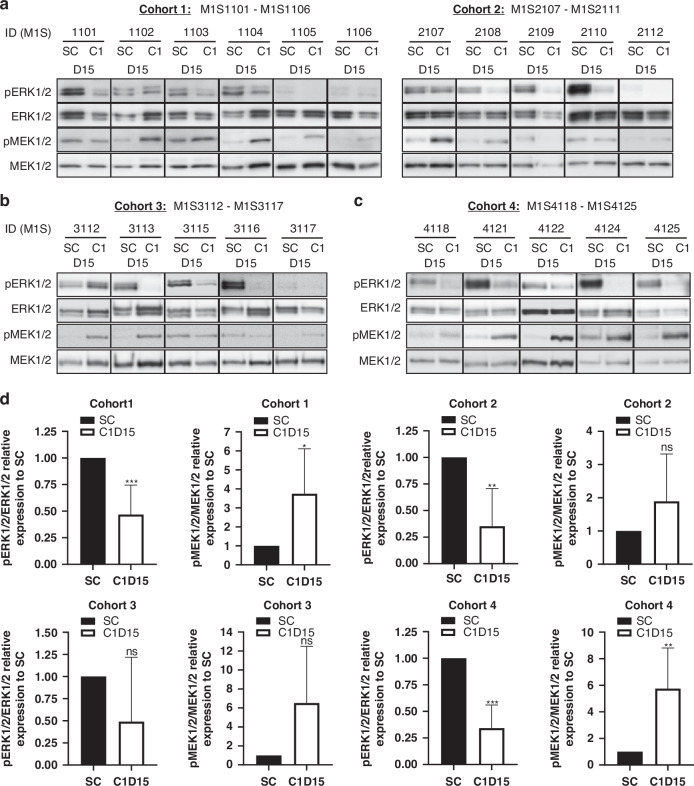
Modulation of pERK1/2^T202/Y204^ and pMEK1/2^S217/221^ expression levels in paired skin biopsies from patients in cohorts 1-4. **a** Cohort 1 and 2: PD-0325901 2 mg B.D. **b** Cohort 3: PD-0325901 4 mg B.D. **c** Cohort 4: PD-0325901 8 mg B.D. **d** Densitometry was performed on the WB images using ImageJ software. SC = screening. C1D15: Skin biopsy obtained between 3 and 6 h following morning dose of PD-0325901.

A total of 21 patients had matched, evaluable pre-treatment and post-treatment skin biopsies to allow evaluation of the PD markers phospho-ERK1/2^T202/Y204^ and phospho-MEK1/2^S217/221^ by Western blotting (Fig. 2). PD-0325901 treatment resulted in a significant accumulation of catalytically-inactive pMEK1/2 [31], in particular in the patients who received 8 mg B.D dose of PD-0325901. Densitometry analyses also showed a marked reduction in pERK1/2 levels following 15 days of combined PD-0325901/crizotinib treatment in all cohorts, but this was only significant in cohorts 1, 2 and 4.

### Efficacy

**Fig. 3:**
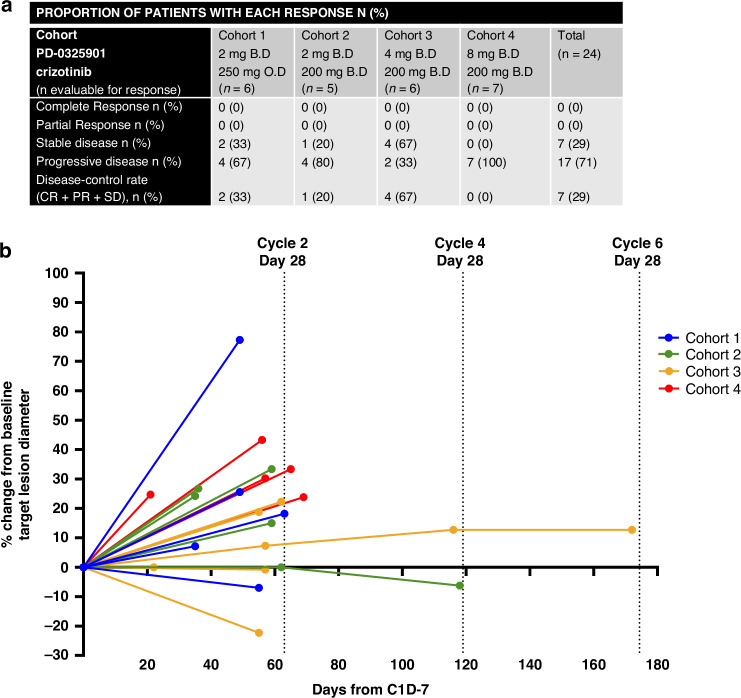
Objectives responses according to RECIST 1.1. criteria. **a** Best radiological response observed to treatment as per cohort. **b** Spider plot of radiological response. Tumour responses were measured and values show percent change of the sum of target lesion diameters from the baseline measurements of each measurable tumour. Changes in target tumour diameter for patients included in cohort 1, cohort 2, cohort 3 and cohort 4 are represented in blue, green, yellow and red respectively.

A total of 24 patients were eligible for response assessment. There were no objective responses to treatment observed, although 7 patients (29%) had radiologically stable disease (Fig. 3a) and one patient had prolonged disease stabilisation for 6 cycles (Fig. 3b). Median PFS on treatment was 1.9 months, and the most common reason for discontinuing treatment was disease progression. Median OS was 6.4 months.

## Discussion

To our knowledge, this is the first study evaluating the safety of a MEK1/2 inhibitor when combined with a c-MET inhibitor. This phase I dose-escalation study met its primary objective of establishing the MTD and assessing the safety and toxicity profile of combined MEK1/2 inhibitor treatment PD-0325901 with c-MET inhibition crizotinib in patients with pre-treated advanced solid tumours.

This phase I dose-escalation study demonstrated that the combination of PD-0325901 with crizotinib is safe, without any unexpected toxicities. The most common adverse events included rash, nausea, diarrhoea, fatigue, vomiting, oedema, anaemia and hypoalbuminaemia, and the combination did not increase the incidence of these toxicities compared with the administration of either agent alone [21, 22, 32, 33]. Furthermore, the DR-AE’s were mostly mild to moderate (≤ grade 2) with an acceptable incidence of grade 3 toxicities in 25% of patients. In particular, visual disturbances were all low grade, without any cases of retinal vein occlusion and, in contrast to the initial phase I/II monotherapy studies with PD-0325901 [21, 22], no cases with neurological toxicity were reported. However, it is likely that the limited number of cycles (median of 2 cycles) and the lower doses of PD-0325901 administered in this study, contributed to the differences in ocular and neurological toxicities reported respectively. One DLT – a grade 3 fatigue on dose level 4 - was observed, and this toxicity was not unexpected as fatigue is known to occur with MEK1/2 and MET inhibitors [21, 32]. Of interest, we did not observe an increase in adverse events when escalating from dose level 1 to 4. On the basis of this study, the MTD was defined to be 8 mg PD-0325901 B.D (days 1–21) and 200 mg crizotinib B.D continuously in a 28-day cycle. Noteworthy, as treatment exposure was limited, long-term tolerability was not assessed in this study.

Concomitant dosing of oral compounds may require a dose adjustment if a PK drug-drug interaction is observed. Therefore, the PK of both crizotinib and PD-0325901 was assessed in the different cohorts within the study and compared with available values from the literature. B.D dosing of crizotinib did result in significant (1.6–2.6 fold) increases in C_max_, C_min_ and AUC_0-10h_ on days 21 and 28 compared with O.D dosing, with values comparable to those published by Tan et al. [27] for crizotinib monotherapy. Additionally, the median trough plasma concentrations of crizotinib observed in all cohorts was in excess of 62 ng/mL, the pre-clinically predicted effective concentration to inhibit c-MET (data not shown) [27]. Importantly, the dose escalation of PD-0325901 between cohort 2 and 4 did not appear to affect the PK parameters of crizotinib measured.

For twice-daily-dosing (B.D) of crizotinib and PD-0325901 (cohorts 2-4), there was a linear relationship between the dose of PD-0325901 and C_max_, C_min_ and AUC_0-10h_ for PD-0325901 and its metabolite PD0315209 on Cycle 1 Day -1 and Day 21 but plasma concentrations for both PD-0325901 and PD-0315209 were lower at Day 21 than at Day -1, in particular in cohorts 2-4. This is in contrast to the previously published PK study from LoRusso et al. [21], which found that with multiple B.D dosing, PD-0325901 showed a slightly higher AUC at day 21 compared to day 1 (first dose). Additionally, when comparing our data for Cycle 1 and 2 for the PD-0325901 8 mg B.D dose only, there was a significant 1.8–2.2-fold decrease in the pre-dose concentrations of PD-0325901 and its metabolite PD-0315209 on Days 21 compared to Day-1. Our data would suggest that, as the multi-dosing of PD-0325901 continues throughout the cycle, the parent drug is either more efficiently metabolised and excreted or less efficiently absorbed. Noteworthy, in contrast to cohort 2, no apparent difference in AUC_0-10h_, C_max_ and C_min_ were observed on Day 21 compared to Day -1 in cohort 1. These data would indicate that crizotinib dosed twice daily has reduced the plasma concentrations of PD-0325901 achieved. Importantly, plasma concentrations of PD-0325901 reached levels consistent with those required to inhibit MEK1/2 activity at all dose levels and inhibited pERK1/2 levels in all 4 cohorts. Taken together, the observed safety profile is not impacted by a PK interaction between the drugs leading to a higher than expected exposure at any of the dose levels tested.

PD-0325901 has previously been combined with other inhibitors of MEK1/2 bypass signalling pathways, showing variable clinical efficacy. In a phase I study of the pan-HER inhibitor dacomitinib with PD-0325901 in patients with advanced *KRAS*MT CRC, lung and pancreatic cancers, Van Geel et al. reported disease stabilisation in 55.5% of evaluable patients, but the combination exhibited significant toxicity [34]. Combined treatment of PD-0325901 with the dual PI3K/mTOR inhibitor gedatolisib, resulted in response rates of 11.1%, but this was in a biomarker-selected (*KRAS* or *BRAF*MT) population [35]. Therefore, the limited anti-tumour activity observed in our clinical trial with crizotinib and PD-0325901 could be attributable to the heavily pre-treated, heterogeneous and biomarker-unselected participants in our study, together with the limited dataset.

In summary, inhibition of the MEK1/2 and c-MET kinases using PD-0325901 and crizotinib, was well tolerated with a manageable toxicity profile, but with limited activity in unselected heavily pre-treated patients with advanced solid tumours. Further exploration of this combination in a biomarker selected population of patients with advanced CRC was not pursued due to the termination of the clinical development of PD-0325901. Moving forward, a phase Ia/b trial evaluating the combination of crizotinib with an alternative MEK1/2 inhibitor binimetinib in *RAS* mutant CRC patients with aberrant c-MET was completed.

## Supplementary information

Supplementary figures and tables

## Data Availability

Requests for use of the individual participant data after publication should be made in writing to the Oncology Clinical Trials Office (OCTO) and will be managed as per contemporaneous applicable data sharing procedures.
