# Supplementary material for: A phase Ia study of the MEK1/2 inhibitor PD-0325901 with the c-MET inhibitor crizotinib in patients with advanced solid cancers
Source: BJC Rep. 2025 Mar 26;3:17. doi: 10.1038/s44276-025-00133-6 (PMC11947101; doi:10.1038/s44276-025-00133-6)
Supplement: Supplementary file 1 — Supplementary figures and tables [file 44276_2025_133_MOESM1_ESM.pptx]

## Slide 1
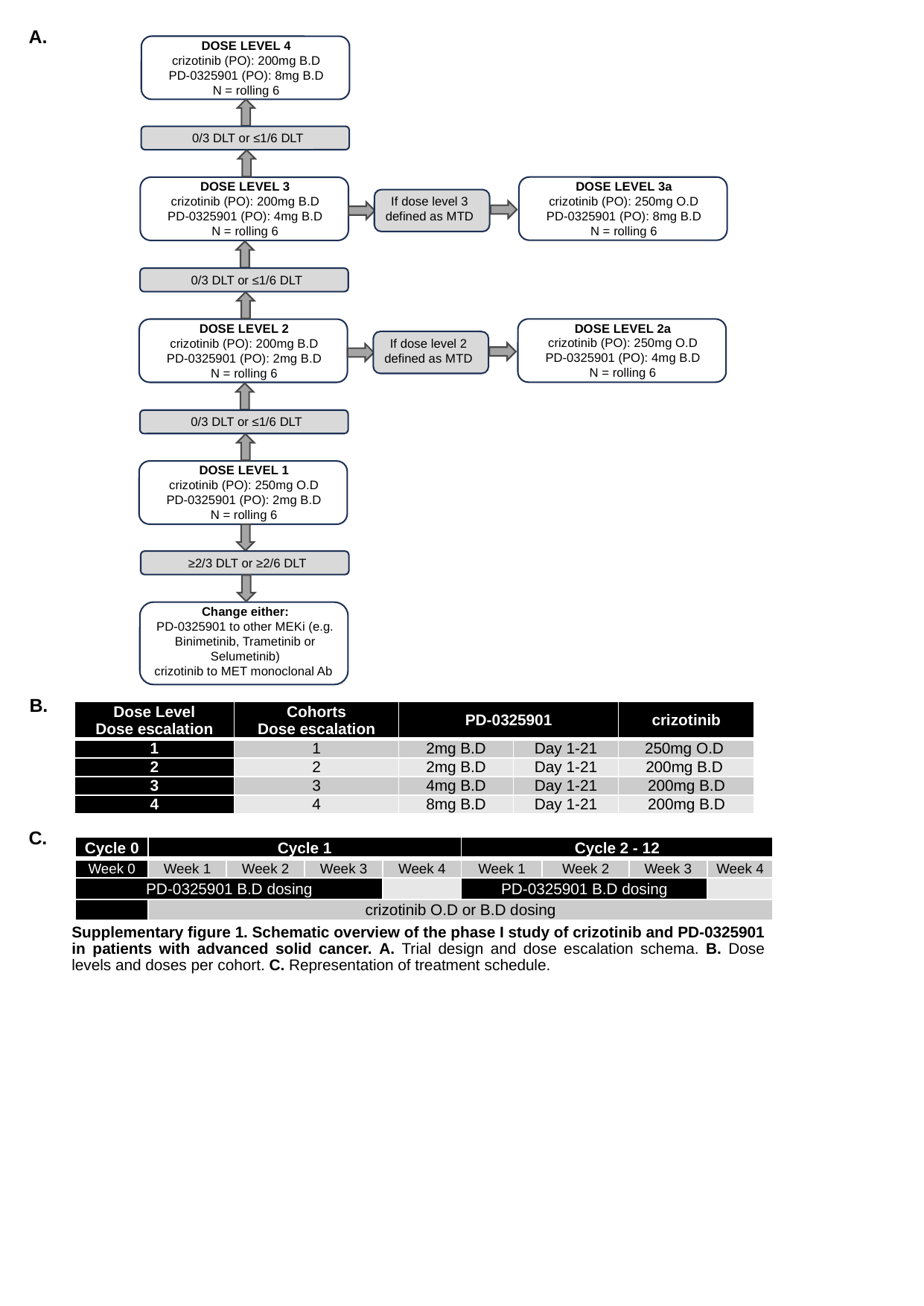

A.
DOSE LEVEL 4
crizotinib (PO): 200mg B.D
PD-0325901 (PO): 8mg B.D
N = rolling 6
0/3 DLT or ≤1/6 DLT
DOSE LEVEL 3a
crizotinib (PO): 250mg O.D
PD-0325901 (PO): 8mg B.D
N = rolling 6
DOSE LEVEL 3
crizotinib (PO): 200mg B.D
PD-0325901 (PO): 4mg B.D
N = rolling 6
If dose level 3 defined as MTD
0/3 DLT or ≤1/6 DLT
DOSE LEVEL 2a
crizotinib (PO): 250mg O.D
PD-0325901 (PO): 4mg B.D
N = rolling 6
DOSE LEVEL 2
crizotinib (PO): 200mg B.D
PD-0325901 (PO): 2mg B.D
N = rolling 6
If dose level 2 defined as MTD
0/3 DLT or ≤1/6 DLT
DOSE LEVEL 1
crizotinib (PO): 250mg O.D
PD-0325901 (PO): 2mg B.D
N = rolling 6
≥2/3 DLT or ≥2/6 DLT
Change either:
PD-0325901 to other MEKi (e.g. Binimetinib, Trametinib or Selumetinib)
crizotinib to MET monoclonal Ab
B.
| Dose Level Dose escalation | Cohorts Dose escalation | PD-0325901 | | crizotinib |
| --- | --- | --- | --- | --- |
| 1 | 1 | 2mg B.D | Day 1-21 | 250mg O.D |
| 2 | 2 | 2mg B.D | Day 1-21 | 200mg B.D |
| 3 | 3 | 4mg B.D | Day 1-21 | 200mg B.D |
| 4 | 4 | 8mg B.D | Day 1-21 | 200mg B.D |
C.
| Cycle 0 | Cycle 1 | | | | Cycle 2 - 12 | | | |
| --- | --- | --- | --- | --- | --- | --- | --- | --- |
| Week 0 | Week 1 | Week 2 | Week 3 | Week 4 | Week 1 | Week 2 | Week 3 | Week 4 |
| PD-0325901 B.D dosing | | | | | PD-0325901 B.D dosing | | | |
| | crizotinib O.D or B.D dosing | | | | | | | |
Supplementary figure 1. Schematic overview of the phase I study of crizotinib and PD-0325901 in patients with advanced solid cancer. A. Trial design and dose escalation schema. B. Dose levels and doses per cohort. C. Representation of treatment schedule.

## Slide 2
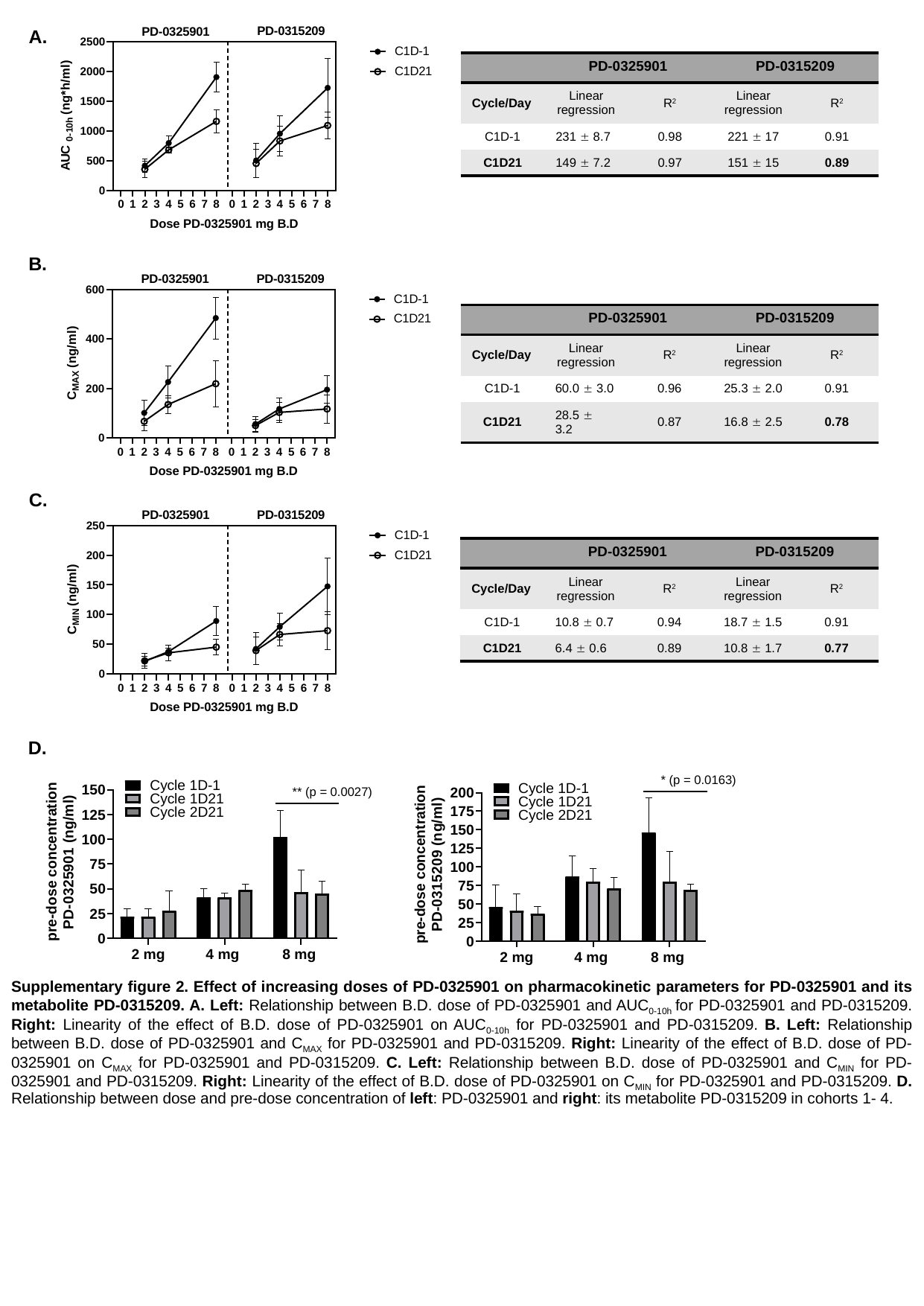

A.
| | PD-0325901 | | PD-0315209 | |
| --- | --- | --- | --- | --- |
| Cycle/Day | Linear regression | R2 | Linear regression | R2 |
| C1D-1 | 231 ± 8.7 | 0.98 | 221 ± 17 | 0.91 |
| C1D21 | 149 ± 7.2 | 0.97 | 151 ± 15 | 0.89 |
B.
| | PD-0325901 | | PD-0315209 | |
| --- | --- | --- | --- | --- |
| Cycle/Day | Linear regression | R2 | Linear regression | R2 |
| C1D-1 | 60.0 ± 3.0 | 0.96 | 25.3 ± 2.0 | 0.91 |
| C1D21 | 28.5 ± 3.2 | 0.87 | 16.8 ± 2.5 | 0.78 |
C.
| | PD-0325901 | | PD-0315209 | |
| --- | --- | --- | --- | --- |
| Cycle/Day | Linear regression | R2 | Linear regression | R2 |
| C1D-1 | 10.8 ± 0.7 | 0.94 | 18.7 ± 1.5 | 0.91 |
| C1D21 | 6.4 ± 0.6 | 0.89 | 10.8 ± 1.7 | 0.77 |
D.
Supplementary figure 2. Effect of increasing doses of PD-0325901 on pharmacokinetic parameters for PD-0325901 and its metabolite PD-0315209. A. Left: Relationship between B.D. dose of PD-0325901 and AUC0-10h for PD-0325901 and PD-0315209. Right: Linearity of the effect of B.D. dose of PD-0325901 on AUC0-10h for PD-0325901 and PD-0315209. B. Left: Relationship between B.D. dose of PD-0325901 and CMAX for PD-0325901 and PD-0315209. Right: Linearity of the effect of B.D. dose of PD-0325901 on CMAX for PD-0325901 and PD-0315209. C. Left: Relationship between B.D. dose of PD-0325901 and CMIN for PD-0325901 and PD-0315209. Right: Linearity of the effect of B.D. dose of PD-0325901 on CMIN for PD-0325901 and PD-0315209. D. Relationship between dose and pre-dose concentration of left: PD-0325901 and right: its metabolite PD-0315209 in cohorts 1- 4.

## Slide 3
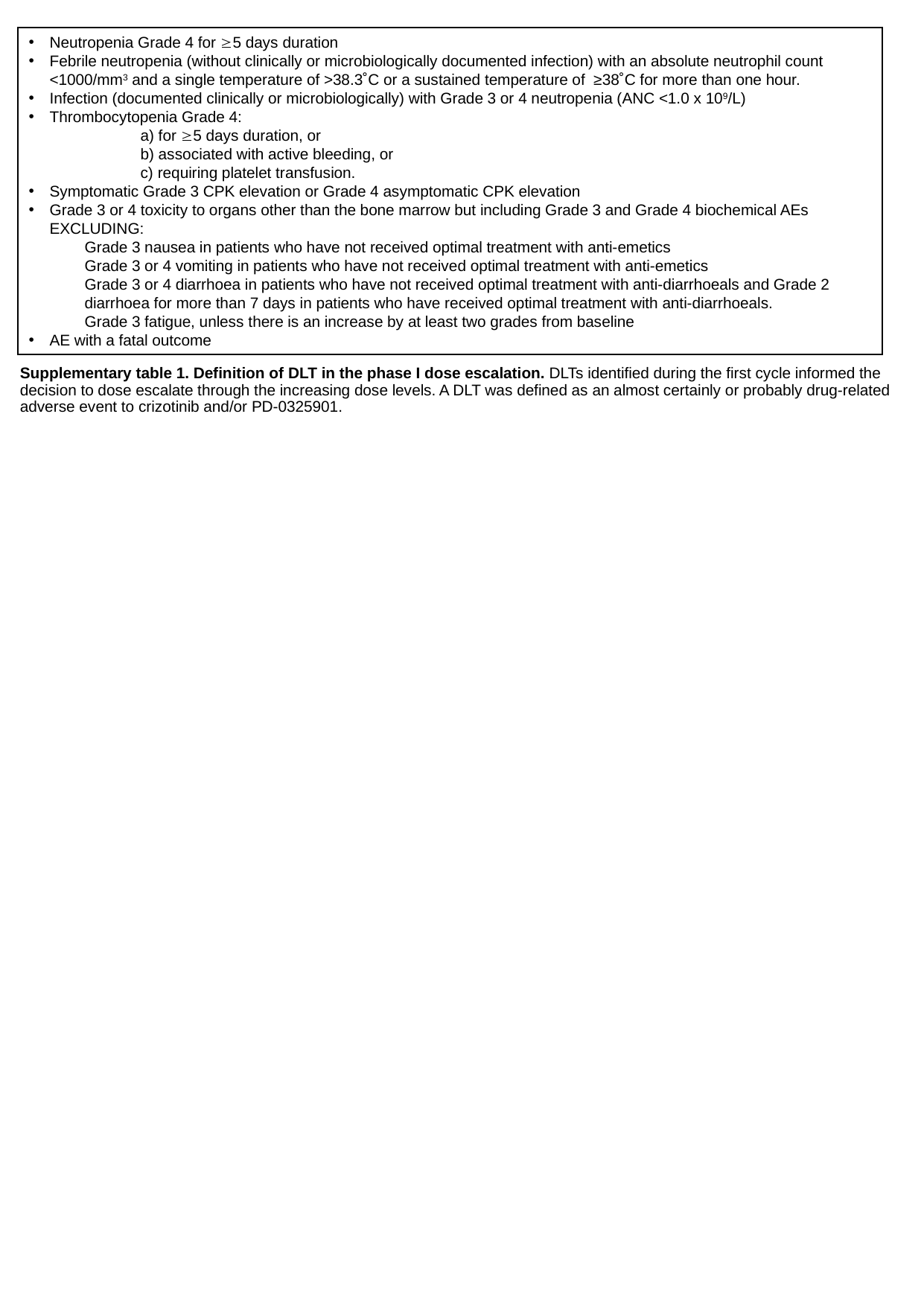

Neutropenia Grade 4 for ≥5 days duration
Febrile neutropenia (without clinically or microbiologically documented infection) with an absolute neutrophil count <1000/mm3 and a single temperature of >38.3˚C or a sustained temperature of ≥38˚C for more than one hour.
Infection (documented clinically or microbiologically) with Grade 3 or 4 neutropenia (ANC <1.0 x 10^9^/L)
Thrombocytopenia Grade 4:
	a) for ≥5 days duration, or
	b) associated with active bleeding, or
	c) requiring platelet transfusion.
Symptomatic Grade 3 CPK elevation or Grade 4 asymptomatic CPK elevation
Grade 3 or 4 toxicity to organs other than the bone marrow but including Grade 3 and Grade 4 biochemical AEs 	EXCLUDING:
Grade 3 nausea in patients who have not received optimal treatment with anti-emetics
Grade 3 or 4 vomiting in patients who have not received optimal treatment with anti-emetics
Grade 3 or 4 diarrhoea in patients who have not received optimal treatment with anti-diarrhoeals and Grade 2 diarrhoea for more than 7 days in patients who have received optimal treatment with anti-diarrhoeals.
Grade 3 fatigue, unless there is an increase by at least two grades from baseline
AE with a fatal outcome
# Supplementary table 1. Definition of DLT in the phase I dose escalation. DLTs identified during the first cycle informed the decision to dose escalate through the increasing dose levels. A DLT was defined as an almost certainly or probably drug-related adverse event to crizotinib and/or PD-0325901.

## Slide 4
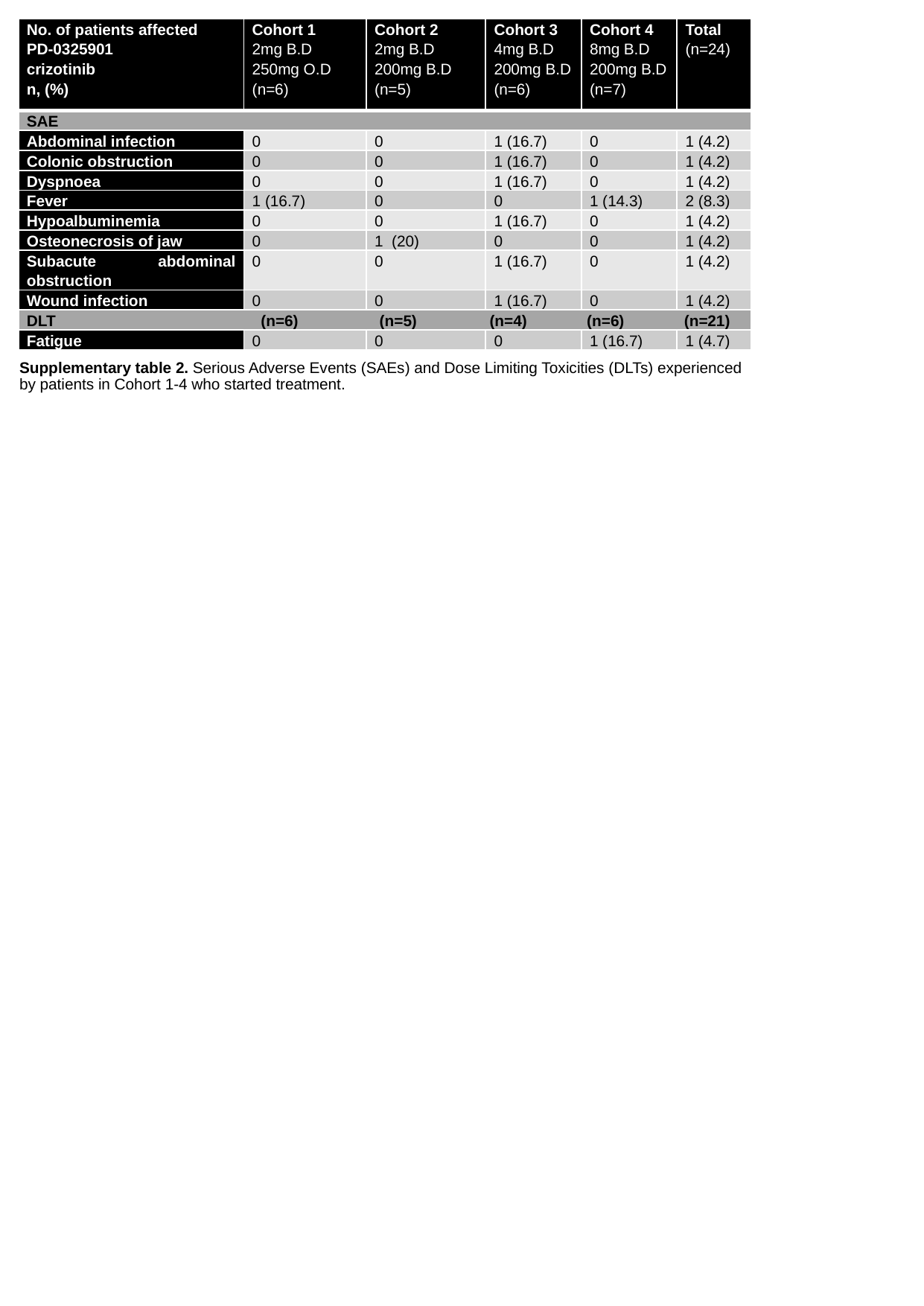

| No. of patients affected PD-0325901 crizotinib n, (%) | Cohort 1 2mg B.D 250mg O.D (n=6) | Cohort 2 2mg B.D 200mg B.D (n=5) | Cohort 3 4mg B.D 200mg B.D (n=6) | Cohort 4 8mg B.D 200mg B.D (n=7) | Total (n=24) |
| --- | --- | --- | --- | --- | --- |
| SAE | | | | | |
| Abdominal infection | 0 | 0 | 1 (16.7) | 0 | 1 (4.2) |
| Colonic obstruction | 0 | 0 | 1 (16.7) | 0 | 1 (4.2) |
| Dyspnoea | 0 | 0 | 1 (16.7) | 0 | 1 (4.2) |
| Fever | 1 (16.7) | 0 | 0 | 1 (14.3) | 2 (8.3) |
| Hypoalbuminemia | 0 | 0 | 1 (16.7) | 0 | 1 (4.2) |
| Osteonecrosis of jaw | 0 | 1 (20) | 0 | 0 | 1 (4.2) |
| Subacute abdominal obstruction | 0 | 0 | 1 (16.7) | 0 | 1 (4.2) |
| Wound infection | 0 | 0 | 1 (16.7) | 0 | 1 (4.2) |
| DLT (n=6) (n=5) (n=4) (n=6) (n=21) | | | | | |
| Fatigue | 0 | 0 | 0 | 1 (16.7) | 1 (4.7) |
Supplementary table 2. Serious Adverse Events (SAEs) and Dose Limiting Toxicities (DLTs) experienced by patients in Cohort 1-4 who started treatment.

## Slide 5
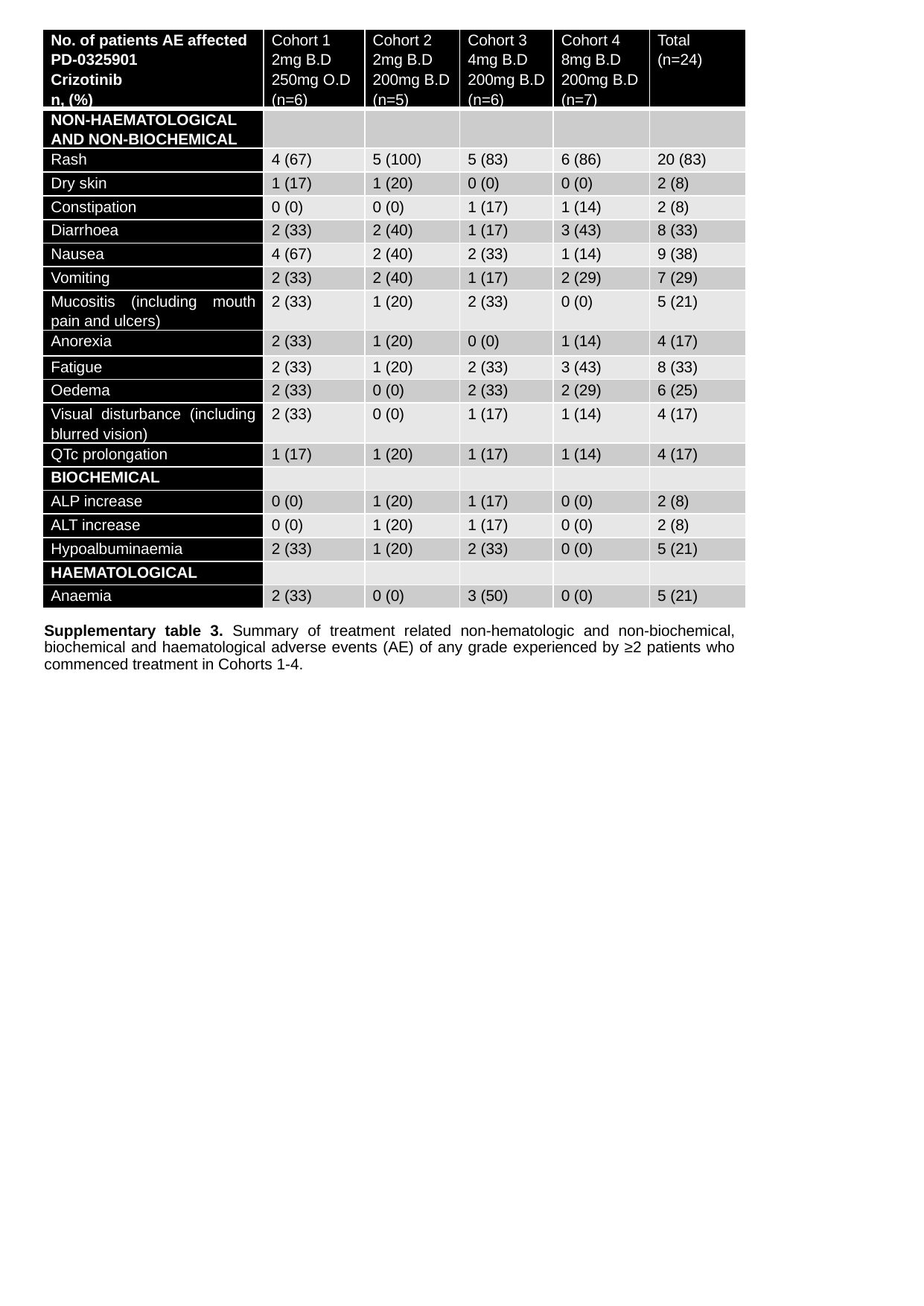

| No. of patients AE affected PD-0325901 Crizotinib n, (%) | Cohort 1 2mg B.D 250mg O.D (n=6) | Cohort 2 2mg B.D 200mg B.D (n=5) | Cohort 3 4mg B.D 200mg B.D (n=6) | Cohort 4 8mg B.D 200mg B.D (n=7) | Total (n=24) |
| --- | --- | --- | --- | --- | --- |
| NON-HAEMATOLOGICAL AND NON-BIOCHEMICAL | | | | | |
| Rash | 4 (67) | 5 (100) | 5 (83) | 6 (86) | 20 (83) |
| Dry skin | 1 (17) | 1 (20) | 0 (0) | 0 (0) | 2 (8) |
| Constipation | 0 (0) | 0 (0) | 1 (17) | 1 (14) | 2 (8) |
| Diarrhoea | 2 (33) | 2 (40) | 1 (17) | 3 (43) | 8 (33) |
| Nausea | 4 (67) | 2 (40) | 2 (33) | 1 (14) | 9 (38) |
| Vomiting | 2 (33) | 2 (40) | 1 (17) | 2 (29) | 7 (29) |
| Mucositis (including mouth pain and ulcers) | 2 (33) | 1 (20) | 2 (33) | 0 (0) | 5 (21) |
| Anorexia | 2 (33) | 1 (20) | 0 (0) | 1 (14) | 4 (17) |
| Fatigue | 2 (33) | 1 (20) | 2 (33) | 3 (43) | 8 (33) |
| Oedema | 2 (33) | 0 (0) | 2 (33) | 2 (29) | 6 (25) |
| Visual disturbance (including blurred vision) | 2 (33) | 0 (0) | 1 (17) | 1 (14) | 4 (17) |
| QTc prolongation | 1 (17) | 1 (20) | 1 (17) | 1 (14) | 4 (17) |
| BIOCHEMICAL | | | | | |
| ALP increase | 0 (0) | 1 (20) | 1 (17) | 0 (0) | 2 (8) |
| ALT increase | 0 (0) | 1 (20) | 1 (17) | 0 (0) | 2 (8) |
| Hypoalbuminaemia | 2 (33) | 1 (20) | 2 (33) | 0 (0) | 5 (21) |
| HAEMATOLOGICAL | | | | | |
| Anaemia | 2 (33) | 0 (0) | 3 (50) | 0 (0) | 5 (21) |
# Supplementary table 3. Summary of treatment related non-hematologic and non-biochemical, biochemical and haematological adverse events (AE) of any grade experienced by ≥2 patients who commenced treatment in Cohorts 1-4.

## Slide 6
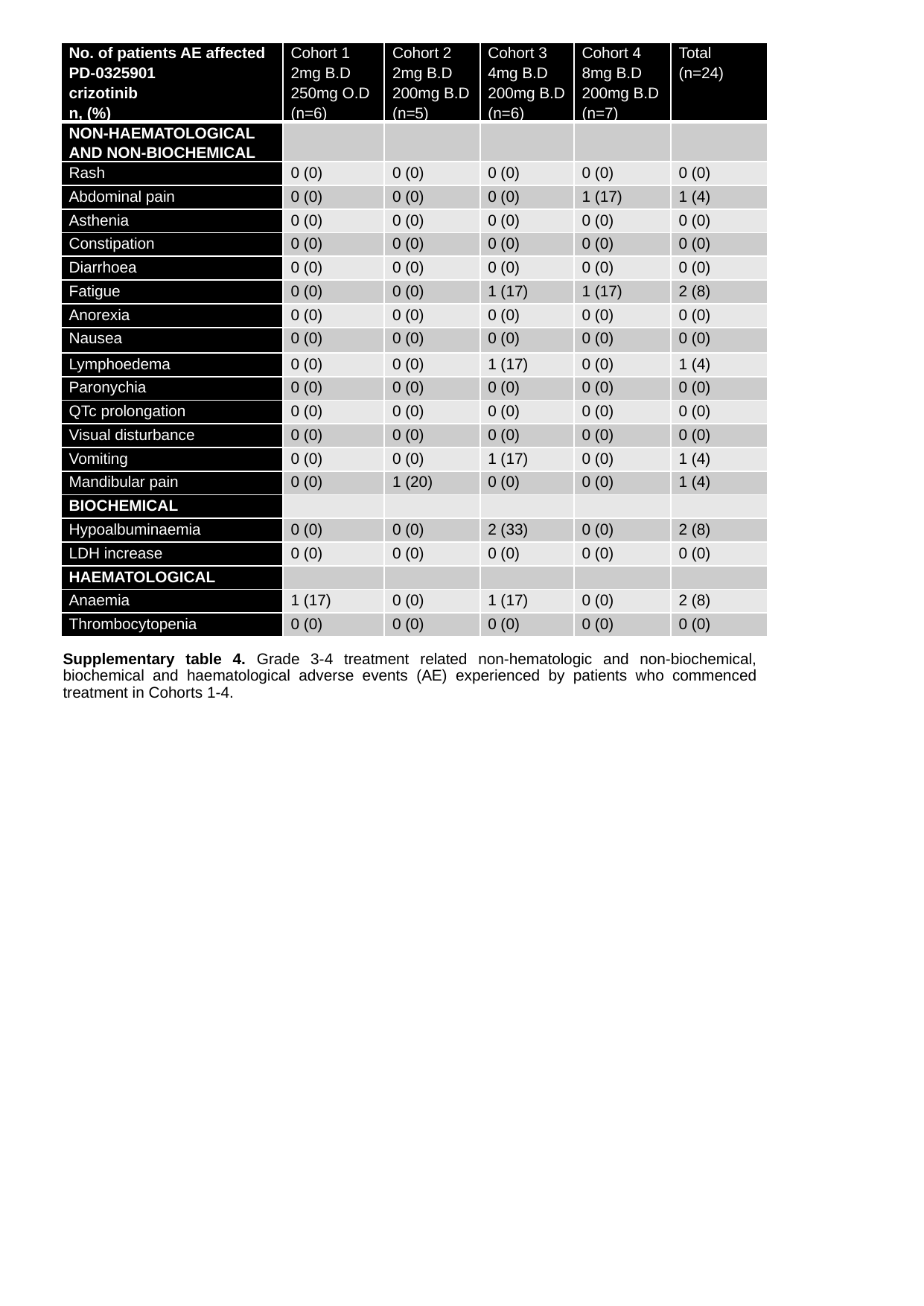

| No. of patients AE affected PD-0325901 crizotinib n, (%) | Cohort 1 2mg B.D 250mg O.D (n=6) | Cohort 2 2mg B.D 200mg B.D (n=5) | Cohort 3 4mg B.D 200mg B.D (n=6) | Cohort 4 8mg B.D 200mg B.D (n=7) | Total (n=24) |
| --- | --- | --- | --- | --- | --- |
| NON-HAEMATOLOGICAL AND NON-BIOCHEMICAL | | | | | |
| Rash | 0 (0) | 0 (0) | 0 (0) | 0 (0) | 0 (0) |
| Abdominal pain | 0 (0) | 0 (0) | 0 (0) | 1 (17) | 1 (4) |
| Asthenia | 0 (0) | 0 (0) | 0 (0) | 0 (0) | 0 (0) |
| Constipation | 0 (0) | 0 (0) | 0 (0) | 0 (0) | 0 (0) |
| Diarrhoea | 0 (0) | 0 (0) | 0 (0) | 0 (0) | 0 (0) |
| Fatigue | 0 (0) | 0 (0) | 1 (17) | 1 (17) | 2 (8) |
| Anorexia | 0 (0) | 0 (0) | 0 (0) | 0 (0) | 0 (0) |
| Nausea | 0 (0) | 0 (0) | 0 (0) | 0 (0) | 0 (0) |
| Lymphoedema | 0 (0) | 0 (0) | 1 (17) | 0 (0) | 1 (4) |
| Paronychia | 0 (0) | 0 (0) | 0 (0) | 0 (0) | 0 (0) |
| QTc prolongation | 0 (0) | 0 (0) | 0 (0) | 0 (0) | 0 (0) |
| Visual disturbance | 0 (0) | 0 (0) | 0 (0) | 0 (0) | 0 (0) |
| Vomiting | 0 (0) | 0 (0) | 1 (17) | 0 (0) | 1 (4) |
| Mandibular pain | 0 (0) | 1 (20) | 0 (0) | 0 (0) | 1 (4) |
| BIOCHEMICAL | | | | | |
| Hypoalbuminaemia | 0 (0) | 0 (0) | 2 (33) | 0 (0) | 2 (8) |
| LDH increase | 0 (0) | 0 (0) | 0 (0) | 0 (0) | 0 (0) |
| HAEMATOLOGICAL | | | | | |
| Anaemia | 1 (17) | 0 (0) | 1 (17) | 0 (0) | 2 (8) |
| Thrombocytopenia | 0 (0) | 0 (0) | 0 (0) | 0 (0) | 0 (0) |
# Supplementary table 4. Grade 3-4 treatment related non-hematologic and non-biochemical, biochemical and haematological adverse events (AE) experienced by patients who commenced treatment in Cohorts 1-4.

## Slide 7
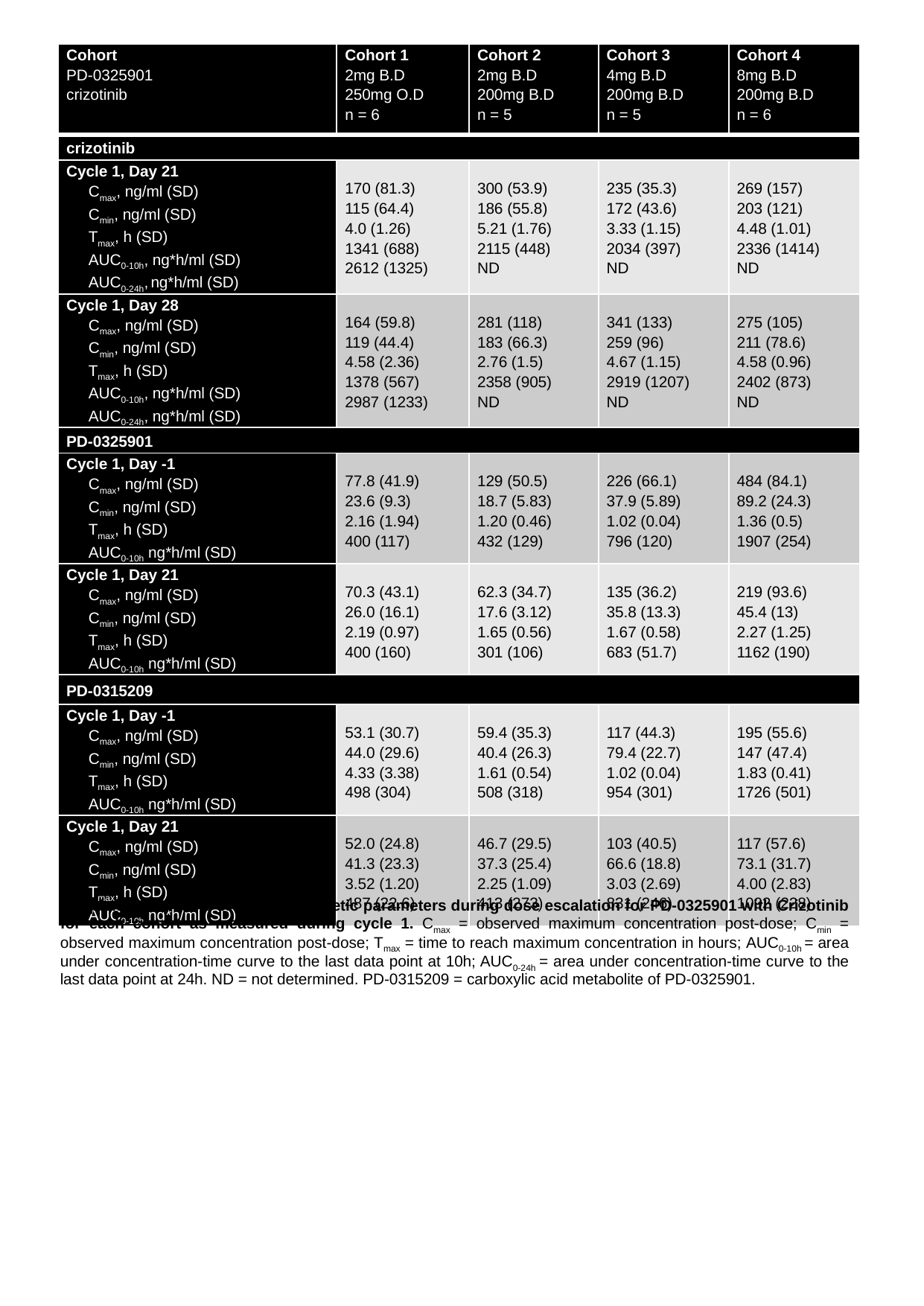

| Cohort PD-0325901 crizotinib | Cohort 1 2mg B.D 250mg O.D n = 6 | Cohort 2 2mg B.D 200mg B.D n = 5 | Cohort 3 4mg B.D 200mg B.D n = 5 | Cohort 4 8mg B.D 200mg B.D n = 6 |
| --- | --- | --- | --- | --- |
| crizotinib | | | | |
| Cycle 1, Day 21 Cmax, ng/ml (SD) Cmin, ng/ml (SD) Tmax, h (SD) AUC0-10h, ng\*h/ml (SD) AUC0-24h, ng\*h/ml (SD) | 170 (81.3) 115 (64.4) 4.0 (1.26) 1341 (688) 2612 (1325) | 300 (53.9) 186 (55.8) 5.21 (1.76) 2115 (448) ND | 235 (35.3) 172 (43.6) 3.33 (1.15) 2034 (397) ND | 269 (157) 203 (121) 4.48 (1.01) 2336 (1414) ND |
| Cycle 1, Day 28 Cmax, ng/ml (SD) Cmin, ng/ml (SD) Tmax, h (SD) AUC0-10h, ng\*h/ml (SD) AUC0-24h, ng\*h/ml (SD) | 164 (59.8) 119 (44.4) 4.58 (2.36) 1378 (567) 2987 (1233) | 281 (118) 183 (66.3) 2.76 (1.5) 2358 (905) ND | 341 (133) 259 (96) 4.67 (1.15) 2919 (1207) ND | 275 (105) 211 (78.6) 4.58 (0.96) 2402 (873) ND |
| PD-0325901 | | | | |
| Cycle 1, Day -1 Cmax, ng/ml (SD) Cmin, ng/ml (SD) Tmax, h (SD) AUC0-10h ng\*h/ml (SD) | 77.8 (41.9) 23.6 (9.3) 2.16 (1.94) 400 (117) | 129 (50.5) 18.7 (5.83) 1.20 (0.46) 432 (129) | 226 (66.1) 37.9 (5.89) 1.02 (0.04) 796 (120) | 484 (84.1) 89.2 (24.3) 1.36 (0.5) 1907 (254) |
| Cycle 1, Day 21 Cmax, ng/ml (SD) Cmin, ng/ml (SD) Tmax, h (SD) AUC0-10h ng\*h/ml (SD) | 70.3 (43.1) 26.0 (16.1) 2.19 (0.97) 400 (160) | 62.3 (34.7) 17.6 (3.12) 1.65 (0.56) 301 (106) | 135 (36.2) 35.8 (13.3) 1.67 (0.58) 683 (51.7) | 219 (93.6) 45.4 (13) 2.27 (1.25) 1162 (190) |
| PD-0315209 | | | | |
| Cycle 1, Day -1 Cmax, ng/ml (SD) Cmin, ng/ml (SD) Tmax, h (SD) AUC0-10h ng\*h/ml (SD) | 53.1 (30.7) 44.0 (29.6) 4.33 (3.38) 498 (304) | 59.4 (35.3) 40.4 (26.3) 1.61 (0.54) 508 (318) | 117 (44.3) 79.4 (22.7) 1.02 (0.04) 954 (301) | 195 (55.6) 147 (47.4) 1.83 (0.41) 1726 (501) |
| Cycle 1, Day 21 Cmax, ng/ml (SD) Cmin, ng/ml (SD) Tmax, h (SD) AUC0-10h ng\*h/ml (SD) | 52.0 (24.8) 41.3 (23.3) 3.52 (1.20) 487 (22.6) | 46.7 (29.5) 37.3 (25.4) 2.25 (1.09) 413 (273) | 103 (40.5) 66.6 (18.8) 3.03 (2.69) 831 (246) | 117 (57.6) 73.1 (31.7) 4.00 (2.83) 1092 (228) |
# Supplementary table 5. Pharmacokinetic parameters during dose escalation for PD-0325901 with Crizotinib for each cohort as measured during cycle 1. Cmax = observed maximum concentration post-dose; Cmin = observed maximum concentration post-dose; Tmax = time to reach maximum concentration in hours; AUC0-10h = area under concentration-time curve to the last data point at 10h; AUC0-24h = area under concentration-time curve to the last data point at 24h. ND = not determined. PD-0315209 = carboxylic acid metabolite of PD-0325901.
